# 866. A new protocol for bacterial identification and carbapenemases by MALDI-TOF and lateral flow assay directly from positive blood culture: MALDI-FAST

**DOI:** 10.1093/ofid/ofad500.911

**Published:** 2023-11-27

**Authors:** D I E G O Josa, Edwin Silva, Juan Pablo Osorio, Paula Hernandez, Isabel Torres, Blanca Quiroga, Deisy martinez, Luz Pescador, Lydy Hurtado, Paola Trujillo, Fabian Cortes

**Affiliations:** Fundación Clínica Shaio, Bogota, Cundinamarca, Colombia; Fundación Clínica Shaio, Bogota, Cundinamarca, Colombia; clinica shaio, bogota, Distrito Capital de Bogota, Colombia; clinica shaio, bogota, Distrito Capital de Bogota, Colombia; Fundación Clínica Shaio, Bogota, Cundinamarca, Colombia; clinica shaio, bogota, Distrito Capital de Bogota, Colombia; clinica shaio, bogota, Distrito Capital de Bogota, Colombia; clinica shaio, bogota, Distrito Capital de Bogota, Colombia; clinica shaio, bogota, Distrito Capital de Bogota, Colombia; clinica shaio, bogota, Distrito Capital de Bogota, Colombia; Clínica Shaio, Bogota, Distrito Capital de Bogota, Colombia

## Abstract

**Background:**

Infectious diseases are a global public health problem, accounting for more than 20% of the mortality burden. *Klebsiella pneumoniae* and *Pseudomonas aeruginosa* caused more than 1.3 million deaths globally in 2019. Antimicrobial resistance (AMR) is a major factor in this mortality. Rapid molecular test for the detection of the blaKPC gene improves treatment time and mortality in patients with bacteremia caused by carbapenem-resistant Enterobacterales, however, the use of molecular biology techniques may not be optimal in multiple resourced care settings limited. Our goal is to perform rapid identification of Gram-negative bacilli and detection of carbapenemases directly from blood cultures using the combination of mass spectrometry (MALDI-TOF) and the lateral flow immunochromatography test.4

**Figure:**
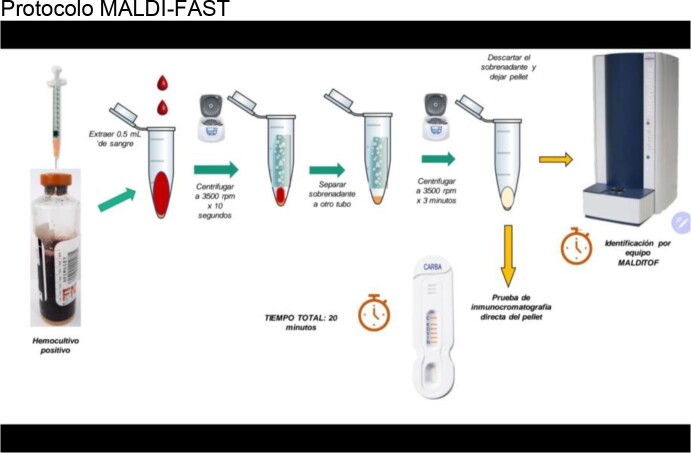

**Methods:**

We analyzed 97 bottles of positive blood cultures detected by the BACT/Alert 3D system (BioMérieux®). Our new method (MALDI-FAST) uses 0.5ml of blood obtained from the positive blood culture bottle for Gram-negative bacilli, from which a pellet is obtained by rapid centrifugation in 3 minutes. This material is processed both for bacterial identification by MALDI-TOF mass spectrophotometry (Bruker®), and rapid detection of carbapenemases by NG Test Carba 5 lateral flow immunochromatography (NG Biotech®). The results obtained were compared with the gold standard RT-PCR FilmArray Panel BCID 2.0 (BioMérieux®)

**Results:**

Of the 97 blood culture bottles, 94/97 (96.9%) were correctly identified to the species level and 3/97 (3.1%) were polymicrobial blood cultures. *Klebsiella pneumoniae* was detected in 39 samples, *E. coli* in 24, and 34 other microorganisms including non-fermenting bacteria. Excellent concordance (kappa index 1,000) was achieved in 31/97 blood cultures (31.9%) that presented carbapenemases, 18/31 KPC (18.6%) and 13/31 with KPC and NDM coproduction (13.4%). 66/97(68.15%) bottles did not present carbapenemase.

**Conclusion:**

Our protocol presents very good results for bacterial identification by mass spectrometry and carbapenemase detection by lateral flow immunoassay in a few minutes directly from the positive blood culture, becoming a fast and cost-effective tool to contribute to patient management.

**Disclosures:**

**All Authors**: No reported disclosures

